# Evidence for Antigenic Seniority in Influenza A (H3N2) Antibody Responses in Southern China

**DOI:** 10.1371/journal.ppat.1002802

**Published:** 2012-07-19

**Authors:** Justin Lessler, Steven Riley, Jonathan M. Read, Shuying Wang, Huachen Zhu, Gavin J. D. Smith, Yi Guan, Chao Qiang Jiang, Derek A. T. Cummings

**Affiliations:** 1 Department of Epidemiology, Johns Hopkins Bloomberg School of Public Health, Baltimore, Maryland, United States of America; 2 School of Public Health, Department of Infectious Disease Epidemiology, MRC Centre for Outbreak Analysis and Modelling, Imperial College, London, United Kingdom; 3 Department of Epidemiology and Population Health, Institute of Infection and Global Health, Faculty of Health and Life Sciences, University of Liverpool, Liverpool, United Kingdom; 4 Guangzhou No. 12 Hospital, Guangzhou, Guangdong, China; 5 International Institute of Infection and Immunity, Shantou University Medical College, Shantou, Guangdong, China; 6 Department of Microbiology, The University of Hong Kong, Hong Kong SAR, China; 7 Laboratory of Virus Evolution, Program in Emerging Infectious Diseases, Duke-NUS Graduate Medical School, Singapore, Singapore; Mount Sinai School of Medicine, United States of America

## Abstract

A key observation about the human immune response to repeated exposure to influenza A is that the first strain infecting an individual apparently produces the strongest adaptive immune response. Although antibody titers measure that response, the interpretation of titers to multiple strains – from the same sera – in terms of infection history is clouded by age effects, cross reactivity and immune waning. From July to September 2009, we collected serum samples from 151 residents of Guangdong Province, China, 7 to 81 years of age. Neutralization tests were performed against strains representing six antigenic clusters of H3N2 influenza circulating between 1968 and 2008, and three recent locally circulating strains. Patterns of neutralization titers were compared based on age at time of testing and age at time of the first isolation of each virus. Neutralization titers were highest for H3N2 strains that circulated in an individual's first decade of life (peaking at 7 years). Further, across strains and ages at testing, statistical models strongly supported a pattern of titers declining smoothly with age at the time a strain was first isolated. Those born 10 or more years after a strain emerged generally had undetectable neutralization titers to that strain (<1∶10). Among those over 60 at time of testing, titers tended to increase with age. The observed pattern in H3N2 neutralization titers can be characterized as one of antigenic seniority: repeated exposure and the immune response combine to produce antibody titers that are higher to more ‘senior’ strains encountered earlier in life.

## Author Summary

The human immune response to an influenza infection is not the same for every infection. It has often been observed that we tend to have the highest antibody titer (and presumably our strongest immune response) against strains of influenza that we were exposed to early in life. In this study, we obtained blood samples from 151 people between 7 and 81 years of age and tested the samples for the concentration of antibodies to many different (H3N2) strains. We chose strains according to when they first circulated, starting with a strain isolated just after the 1968 pandemic and going all the way through to very recent strains. We found that a participant's age at the time a strain first circulated was very predictive of the strength of their antibody against that strain. Not just for the first strain they were likely to have seen, but also for the second, third and all subsequent strains circulating during their lifetime. This suggests to us that antibody titers to influenza A H3N2 follow a pattern of antigenic seniority, suggesting that we produce progressively fewer specific antibodies to each subsequent infection as we age.

## Introduction

It has long been know that humans have a higher serologic response to stains of influenza strains early in their lives, even after vaccination or exposure to more recent strains [1]–[3]. Consistent with this phenomenon, some experimental studies in animals and humans have shown that a second vaccine (or infection) boosts the serological response to earlier infections and may result in a less robust serological response itself [4]–[6]. However, there is some question as to whether this apparent primacy of initial antibodies in a first infection represents greater protection against similar strains and reduced protection against later strains [7]. Little is known about how the relationship between the antibody response to earlier and later infections plays out in the complex patterns of influenza infection and vaccination experienced by real populations. Understanding these patterns may aid in the interpretation of serological evidence (i.e., seroepidemiology), and provide insight into how our immune system interacts with an ever changing pathogen.

The concentration of antibodies associated with different influenza strains is most often determined using the hemagglutination inhibition (HI) or viral neutralization (NT) assay [8]. However, the picture of historic influenza infections offered by these assays is imperfect. Both HI and NT assays only measure the ability of a person's serum to interfere with the processes necessary for viral replication, and do not distinguish between highly specific and cross reactive antibodies [8]. Accurately characterizing how antibody levels change over a lifetime of influenza exposure can aid in the interpretation of serological assays and expand our understanding of how the immune system responds to a complex and ever changing pathogen.

Since they emerged in 1968, human influenza A H3N2 virus strains have been in continual global circulation. During this time, H3N2 strains have undergone continual genetic drift, with genetically similar viruses predominating for one or two seasons before receding [9]. Antigenic drift of these strains is thought to be faster than genetic drift, characterized by clustering of strains within antigenic space and occasional longer jumps to form new clusters [10]. Seasonal H1N1 strains re-emerged in 1977, developing their own sequential lineage, and continue to co-circulate with H3N2 strains to the present [11]. Nonetheless, H3N2 strains represent a sustained lineage with rapid and regular turn-over of genetically and antigenically distinct strains. As such, they present an opportunity to explore the relationship between the birth-year of individuals and their antibody response to key strains, each of which represents a possible exposure or infection at a different time in an individual's life. While it may not be possible to know exactly which strains each individual was infected with, the combination of titer and age may give us some insight into each individual's history of infection. Across an entire population, the relationship between age of potential infection and titer may reveal patterns that increase our understanding of influenza biology and our ability to interpret serological surveys.

Here we characterize the serologic profiles to historic strains of H3N2 influenza in a population from Guangdong province, China. We develop a statistical model characterizing the relationship between age and neutralization titers to strains of H3N2 influenza circulating from 1968–2008. We propose a refinement of the original antigenic sin hypothesis, antigenic seniority, which may better explain the patterns of immune response seen in this population.

## Results

### Participant recruitment

Of 273 participants interviewed, 151 provided serum and were tested for H3N2 antibodies. Samples were more often from adults than children (Table 1). Age at time of testing ranged from 7 to 81 years of age. Age at time of strain isolation ranged from 34 years before birth (for A/Hong Kong/1968 (H3N2)) to 80 years of age (for A/Shantou/2008 (H3N2)).

**Table 1:** Demographics and history of vaccination and recent illness among study participants.

	Provided Sample?			
	*Yes N(%)*	*No N(%)*	*p*	*Total N(%)*
*Total*	**151**	122		273
*Sex*				
Male	**82 (54)**	58 (48)	0.30	140 (51)
Female	**69 (46)**	63 (52)		132 (48)
*Age in years*				
<10	**5 (3)**	16 (13)	0.03	21 (8)
10–19	**16 (11)**	11 (9)		7(10)
20–29	**20 (13)**	11 (9)		31(11)
30–39	**24(16)**	19(16)		43(16)
40–49	**26(17)**	9(7)		35(13)
50–59	**27 (18)**	21 (17)		48(18)
60–69	**17 (11)**	19 (16)		36 (13)
70+	**16(11)**	15(12)		31(11)
*Time since last influenza vaccination*				
<1 year	**6 (4)**	11 (9)	0.02	17(6)
1 year	**1 (1)**	3 (2)		4 (1)
2–5 years	**6 (4)**	2 (2)		8 (3)
>5 years	**19 (13)**	6 (5)		25 (9)
Never	**100 (66)**	72 (59)		172 (63)
Unsure/Unknown	**19 (13)**	26 (21)		45 (16)
*Symptoms in Past Month*				
Fever	**5 (3)**	6 (5)	0.49	11 (4)
ILI	**3 (2)**	4 (3)	0.73	7 (3)

P-values are based on a chi-squared test for differences between the distribution of those who did and did not provide a blood sample. Only those who provided a sample are included in the present analysis.

### Description of titers

**Figure 1:**
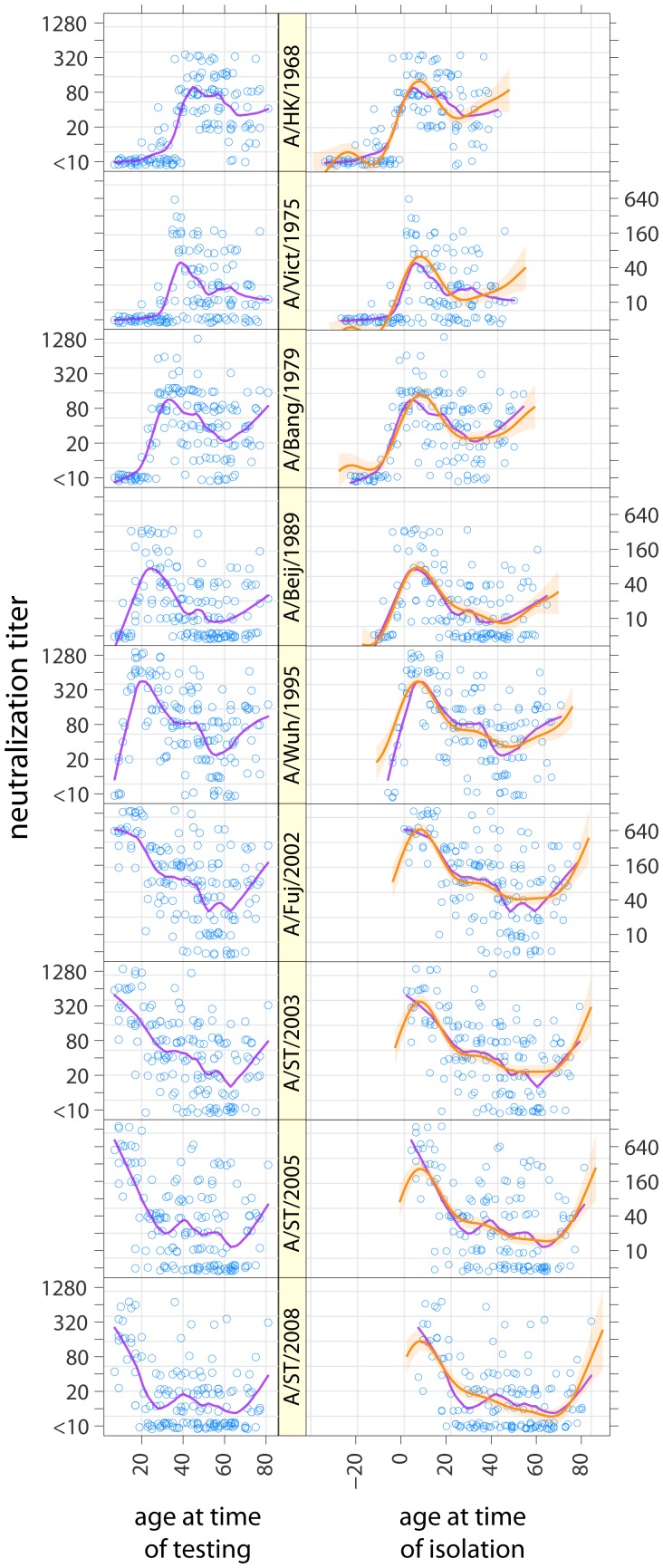
Neutralization titers to strains of H3N2 from 1968 to 2008 by age at time of testing (left) and age at time of isolation of each of the viruses tested (right) (i.e., the right panel shows values in the left panel age shifted by year of strain circulation). Purple lines show smoothed results (LOESS curves, span = 0.25, Gaussian distribution family). Orange lines and shaded regions show predictions and 95% confidence intervals from a log-linear model of neutralization titers where the effect of age at time of isolation and age at time of testing is the same across strains.

**Figure 2:**
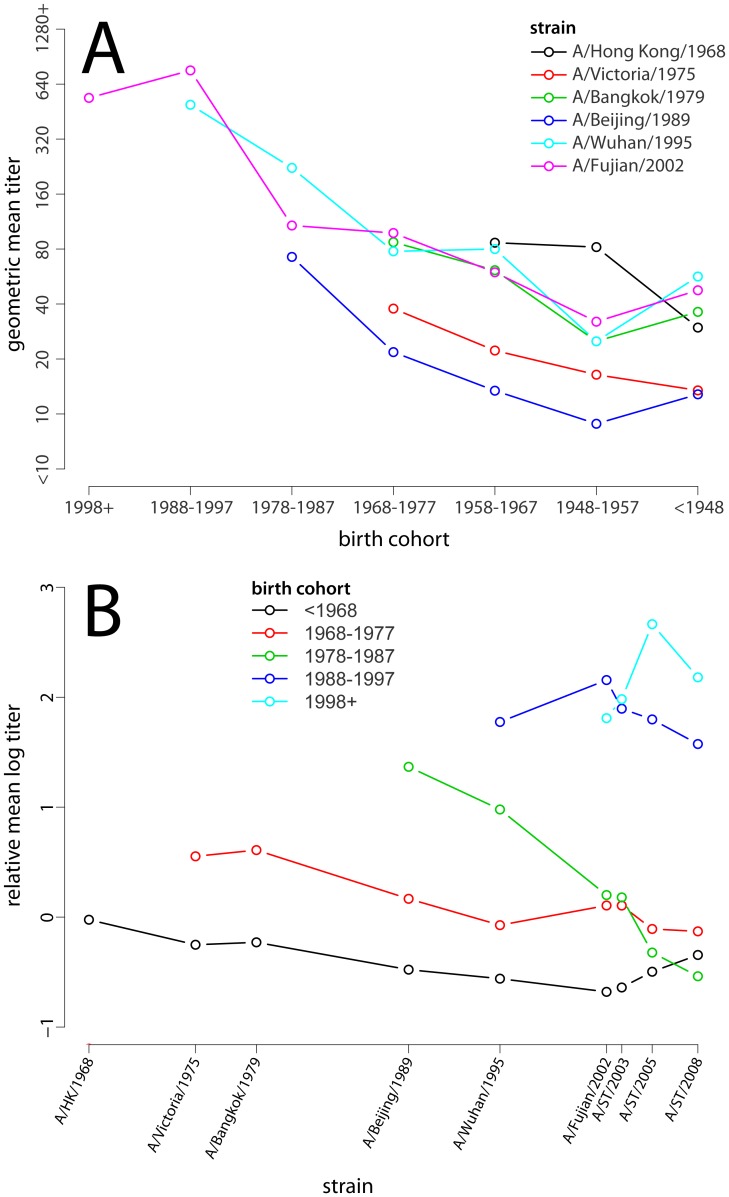
The relationship between birth cohort and mean log titer. (A) Geometric mean of neutralization titer for each strain by birth cohort. Points are only shown if more than half of the birth years in the indicated range occurred after strain isolation (i.e., most of that cohort was alive to be exposed to the given strain). (B) Relative mean log-titer of the given birth cohort compared to all those old enough to be exposed to the given strain. Note that the pattern of increasing maximum titer by birth cohort is a result of the larger pool of individuals with which they are compared (e.g., the <1968 birth cohort is only compared with itself for A/Hong Kong/1968, while the 1978–1987 cohort is compared to all of those born before 1989 for A/Beijing/1989). Points are only shown if more than half of the birth years in the indicated range occurred after strain isolation.

A total of 1,359 (9 strains
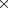
151 individuals) neutralization tests were performed. While peak neutralization titer varied by strain, age-specific mean log-neutralization titers (estimated from a smoothing spline) were consistently highest among those who were in the first decade of life at the time when a given strain was isolated (Figure 1, S1). When we compare the mean log-neutralization titer of all those in a given birth cohort to a given strain, we see that the highest titers for a given strain occur in among the youngest birth cohort alive when that strain was isolated and declines for progressively older cohorts (Figure 2A), and that a birth cohort's titer is highest relative to other birth cohorts for the strains isolated when they were youngest, and declines smoothly for strains isolated later (Figure 2B). In all strains we observe smoothly declining mean titers with increasing age at time of circulation until we reach that cohort of individuals who were 60 or older at the time of sample collection. For those aged 60 and older at the time of sample collection we observe a smooth increase in mean titers with age, seemingly regardless of strain (Figure 1). Those not yet born at the time of strain isolation show the lowest titers to every strain, with most born 10 years or more after strain isolation having neutralization titers below the detectable threshold (<1∶10). For example, against A/Beijing/1989 (H3N2) all those born 10 or more years after 1989 have undetectable titers, those born 0–9 years after 1989 mean log titer of 3.1 (95% CI: 2.3,3.9), those aged 0–9 in 1989 have mean log titers of 4.5 (95% CI: 3.9, 5.0), those aged 10–19 in 1989 have mean log titers of 3.4 (95% CI: 2.9, 3.8), those 20 or older in 1989 but under 70 in 2009 have mean log titers of 2.4 (95% CI: 2.1, 2.6) and those 70 or older in 2009 have mean log titers of 2.7 (95% CI: 2.2, 3.3) (Figure S1).

### Comparison of statistical models of age and titer

We find evidence supporting a strain independent relationship between log neutralization titers, age at time of strain isolation and age at time of testing. To test the hypothesis that there is a common relationship between titer and age, we compared generalized additive models where (A) the relationship between neutralization titer and age is dependent only on the age at time of strain isolation and age at time of testing (relative BIC 0.0) and (B) the relationship between neutralization titer and age (at time of testing) is unique to each strain (relative BIC 206.4). Based on BIC we found model A to be the best model of neutralization titers, and the performance of models A and B was roughly equivalent on other metrics (AICc, performance on held out data, performance of bootstrapped models). We considered two additional generalized additive models that capture the possible role of inherent inter-individual variation in antibody response: (C) a model where each individual has a random intercept and there is a common effect of age at time of strain isolation (relative BIC 542.9) and (D) a model with individual random intercepts and a strain specific effect of age (relative BIC 654.3). While these models are inferior to model A if compared by BIC, they are superior in other measures of model fit (AICc and mean square error from cross validation). However, unlike models A and B, these models cannot be used to predict the titers of individuals outside of the training set. For all models the residuals for log-titers were normally distributed with a standard deviation of approximately one (1.15 for model A, 0.91 for model D) (Figure S3).

Based on its superior BIC and its otherwise equivalent performance to model B, we take model A as the primary model for the remaining analysis. We will refer back to the individual intercept models (C and D) when appropriate.

### The effects of strain and age on neutralization titer

**Figure 3:**
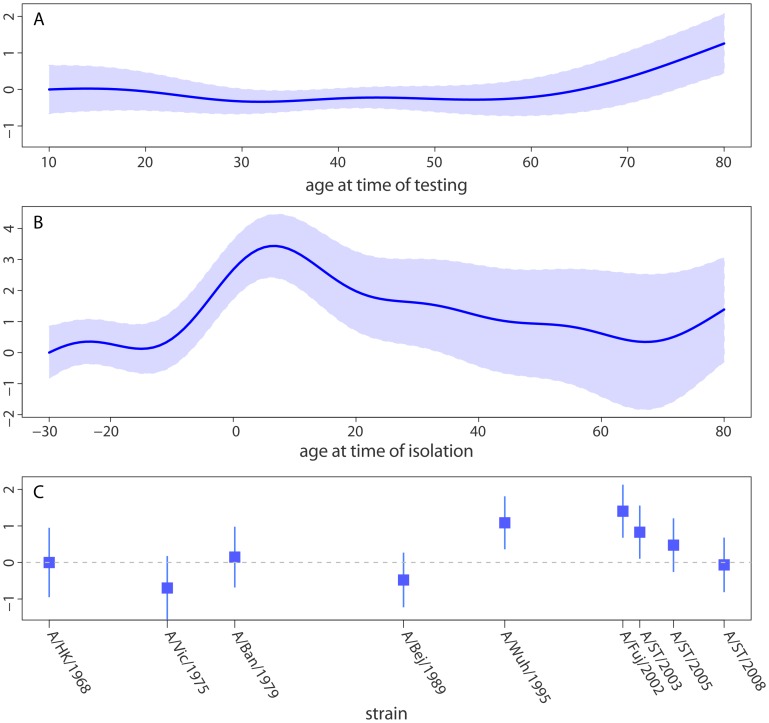
Statistical model of neutralization titers. Strains have shared distributions for the effect of (A) age at time of testing (range 10 to 80, data ranges from 7 to 81) and (B) age at the time when the given strain was isolated (range −30 to 80, data range −34 to 80). Each strain has an independent intercept (C). Relative log titers show the linear change in log neutralization titers compared to the model prediction for the (A) lowest age at time of testing, (B) lowest age at time of strain isolation and (C) A/Hong Kong/1968 (the first strain isolated).

Decomposing the three components of model A (titer by age at time of testing, by age at time of strain isolation and the strain specific intercept) illustrates the effect each of these components has on mean log neutralization titer (Figure 3). Age at testing has little effect on neutralization titer until around age 60, at which point neutralization titers increase smoothly with age (Figure 3A). Age at time of strain isolation causes the largest variation in neutralization titers, with titers peaking at 7 years of age (increase in log titer of 3.4 over baseline, 95% CI, 2.4–4.5) and declining smoothly thereafter (Figure 3B). Those born 10 or more years after a strain was first isolated had the lowest titers to that strain, with increasing titers in those born shortly after or shortly before strain isolation until the 7 year peak. Even after adjusting for the effect of age at time of testing and age at time of isolation there is still variation in titer between tested strains of H3N2, with the highest neutralization titers being seen against A/Fujian/2002 (H3N2) and the lowest against A/Victoria/1975 (H3N2) (Figure 3C). Visual comparison of predictions from this model with log neutralization titer by age at time of isolation show substantial agreement, and confirm the strain independent relationship between age and titer (Figure 1).

**Figure 4:**
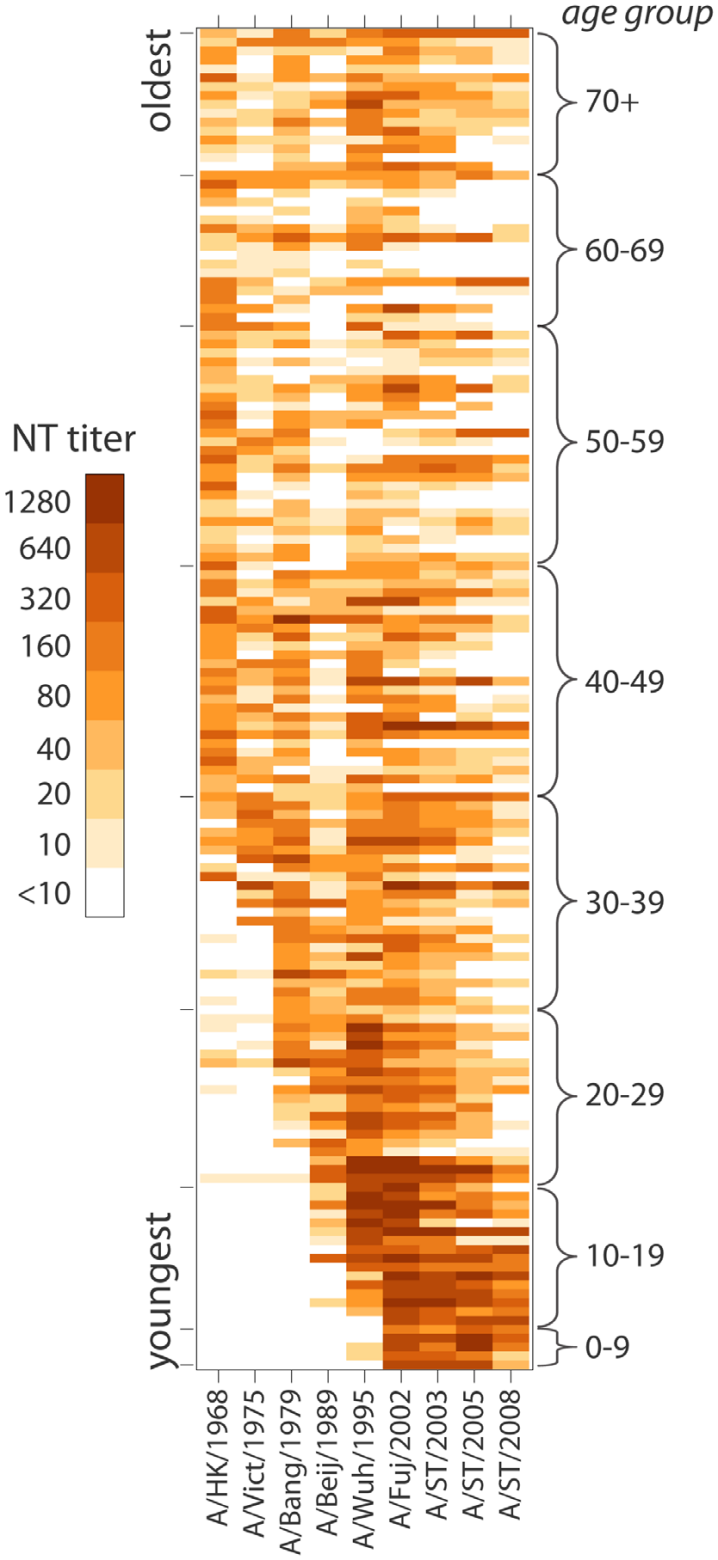
Heat-map of neutralization titers from all 151 individuals to each of the strains tested. Each individual's titers are represented as a row, and individuals are sorted from youngest (on bottom) to oldest (on top). Darker shading indicates a higher neutralization titer.

Examination of each individual's neutralization titer against each tested strain gives further evidence of age-specific patterns in neutralization titers and strain-to-strain variation (Figure 4). Some strains have a very low rate of detectable titers in some age groups. For instance, only 41% of those 50–69 years show neutralization titers 1∶10 (18/44) or higher to A/Beijing/1989. In contrast, 82% (36/44) had titers of 1∶10 or higher to A/Bangkok/1979 (the previous chronological strain among those tested), and 82% (36/44) had titers of 1∶10 or higher to A/Wuhan/1995 (the next chronological strain among those tested).

Despite the importance of age in predicting neutralization titers, individual deviations were common, perhaps attributable to differences in exposure history (Figure 4). For instance, those 60–69 years old generally have undetectable titers (<1∶10) to H3N2 strains isolated 2003 or later, but two individuals (from separate study sites) have high titers to these strains. In model C we account for individuals with generally high or low neutralization titers, strain to strain variation and the age at time of strain isolation; still, 7.7% of measured titers are at least four times higher than predicted and 7.1% are at least four times lower. Models with individuals intercepts outperform other models on metric that do not penalize extra parameters as aggressively as BIC, indicating a possible role for inherent individual variation in antibody response or frequency of influenza exposure not captured by other covariates. However, the maximally complex model has similar performance to the far simpler model A throughout much of the data (Figure S4).

### Comparison with models of Original Antigenic Sin

We compared the performance of model A with models capturing the hypothesis of original antigenic sin. In these models titer depends on a strain intercept and antigenic distance from the first strain in our data that circulated in each participant's lifetime (as measured in by Smith et al., 2004 [12]). Of the models considered (see Supplemental Text S1) the best model of original antigenic sin treated antigenic distance as a linear term and included a terms for whether the participant was alive when the strain circulated. Despite the relative simplicity of this model, this model was out performed by model A in terms of BIC (relative BIC = 34.3). Models of original antigenic sin fit titers for strains circulating before birth well, suggesting that log-neutralization titer decreases by approximately 0.1 log for every unit of antigenic distance from the first possible infecting strain.

### Sensitivity analyses and model validation

Because of missing low vaccination rates and the frequency of missing vaccination status (Table S2), we did not consider vaccination as a covariate in the main analysis. Only 32 of the 151 individuals in our study reported ever having received an influenza vaccination and, of these, only 13 reported receiving vaccination within the last 5 years (Table S2). We performed additional analyses to assess the possibility that vaccination confounded our results. First, we refitted the models using data only from those who reported having never received a vaccination (n = 100) (Figure S5). Second, we refitted the models with an additional binary term for the subset of participants who reported whether or not they had ever received a vaccination (n = 132). Third, on the same subset, we included an additional categorical term capturing the full range of reported vaccination histories (n = 132). None of these analyses produced qualitatively different results.

We performed several tests of model generalizability. First we performed cross validation leaving each titer out from the training data in turn, and then predicting that value using a model fit to the remaining data. Model A performed similarly on cross validated data as to models fit to the full data, while models B, C and D showed small increase in mean squared error (MSE) (Table S1). Second, we fit 500 models to separate bootstrap datasets and used these models to predict on the original data (a technique that is not valid for the individual intercept models, C and D); MSE for the bootstrapped models was similar to that seen when fitting and predicting on the full data (Table S1).

Third, we fit model A with titers for each strain left out in turn. We found that the relationship between titer and age was qualitatively similar regardless of which strain was held out, with the exception of the model fit without A/Hong Kong/1968 (Figure S6). In addition to A/Hong Kong/1968's unique position in our data (it is the first pandemic strain), the model fit excluding this strain does not generalize well to predicting A/Hong Kong/1968 titers and does not increase model fit to other strains (Table S3). For all other strains, the reduced models perform well in predicting the titers of the held out strain, resulting in an increase in mean squared error of 10% or less (Table S3).

Finally, we fit model A with observations for each of the five locations left out in turn. We found that the relationship between titer and age was qualitatively similar regardless of which location was held out, and that the model predicted well on the held out locations (Table S4).

## Discussion

In this study we have demonstrated a clear relationship between the age at first potential exposure to a strain of influenza A (H3N2) and an individual's neutralization titer to that strain. Using robust statistical techniques, we have demonstrated that this age dependence is consistent across strains. Titers are highest against strains circulating when individuals are 5–10 years of age, and then decline steadily thereafter. Independent of strain and age at first potential exposure, titers start to rise after age 60 at time of testing.

The clinical and epidemiological implications of this phenomenon depend on the mechanism leading to these differences. Several plausible options present themselves: immune boosting and interference, age dependent patterns of exposure and changes in the immune system as we age. Immunologic boosting and interference, the tendency for later infections to boost antibody levels to earlier infecting strains and for antibodies to earlier infecting strains to mitigate the later immune response, has historically received the most attention and is supported by experimental evidence [4], [5], [12], [13]. However, it seems that age-specific patterns of influenza infection must also play a role. We observe numerous deviations from the age-specific patterns of neutralization titers that are most easily explained by differences in exposure; and the relationship between age at time of circulation and neutralization titer observed is remarkably similar to the pattern of infection predicted in studies of social contact and mixing patterns [14]. Regardless of the mechanism, it is evident that there remains substantial individual variation in neutralization titer not explained by any of the models considered. This is not unexpected as influenza infection and immune response are influenced by stochastic events which will not be captured by any model.

The peak in neutralization titers to strains circulating when a child is around seven years of age is consistent with recent work showing that children in the Netherlands are infected with at least one strain of influenza A by age seven [15], particularly if (as suggested by the hypothesis of original antigenic sin) the antibody response elicited by this first infection is greater than that elicited by later infections. However, it seems that the patterns observed here are not merely the primacy of the first infecting strain (i.e., original antigenic sin) plus cross-reactivity, as we would then expect that the relationship between age at time of strain isolation and antibody titer would be symmetric around the peak (such an interpretation would also be inconsistent with the experimental results of St Groth et al. [4]). However, there is some reactivity to particular strains among those who were not yet born when the strain circulated. For instance, some individuals born 10 years or more after A/Hong Kong/1968 circulated have titers 1∶20 or greater to that strain. The extent to which those who were not yet born respond to an earlier strain must represent the antigenic similarity between that strain and the ones they were exposed to.

While some studies of immune response post-vaccination suggest that the inhibiting effects of earlier exposures on the production of vaccine strain specific antibodies may have been overstated, [7] the results of numerous population based and experimental studies (including our own) consistently show evidence of an elevated response to the first strains (potentially) encountered. [2]–[4], [16] Additional laboratory experiment and observational work is needed to resolve these discrepancies.

The mechanism behind the apparent increase in antibody titer with age among those over 60 years old at time of testing is unclear. Particularly interesting is the fact that this phenomena is evident in response to strains circulating when these individuals were young, middle aged and old; hence it is unlikely to be explained solely by increased exposure in older individuals. Increased longevity among those with high antibody titers (survivor bias), or the effect of having lived through two influenza pandemics prior to 2009 (60 year-olds would have been school aged in 1957) are both plausible explanations. This former hypothesis is not without precedent, a strong association between higher antibody response and increased lifespan (with death due to causes other than infection) has been observed in experimental mouse models [17], [18].

There are several possible reasons for the strain-to-strain variation that remained even after age at time of testing and age at first circulation is taken into account. These include differences in the extent to which each strain circulated in the region, the intrinsic ability of a specific strain to elicit an immune response, and differences in the neutralizing ability of viral stocks generated for the assays. While residual confounding of the relationship between strain and age is possible, this relationship should be captured by the spline term for age at time of strain isolation.

This study was conducted in five communities in one southern province of China, where exposure histories are likely correlated. Patterns of immune response seen here may be unique to the region, though apparent similarities to historical work suggest that this is not the case [1], [2]. The youngest and oldest age groups are poorly represented: hence, our results may not be generalizable to young children and those over 80. At the extremes of the range of ages seen in the data, the predicted relationship between titer and age will be more sensitive to outliers and may be biased; however, cross-validation results indicate this is likely not the case. Because this was a cross sectional study, it is difficult to identify potential mechanisms behind the pattern of neutralization response to historic H3N2 strains. Knowing an individual's history of influenza infection would aid greatly in the interpretation of our results, but such data requires long running longitudinal observations and is not available in the current cross sectional study. While there may be some differences in the persistence of antibodies by strain, robustness of a model where titer patterns are shared across strains (model A) suggest this is not the case. However, there is some indication of inter-individual variation that may be due to different rates of antibody decay between individuals.

Understanding how serological presentation varies by age has important implications for studies relying on sero-epidemiology. If we hope to measure variations in incidence between populations, it is important that we understand how differences in the age composition of different populations affect observed titers. In vaccine trials, where serological response is used as an immune correlate, understanding the background patterns in influenza serologies can improve the interpretation of results. To the extent that neutralization titers are correlates of influenza immunity, they may have clinical and public health implications. Age-specific patterns of protection may indicate those groups that would benefit most from vaccination or the use of a high antigen vaccine. Correct estimates of age-specific patterns of protection can improve simulation studies aimed at predicting the impact of influenza infection.

The patterns observed here are similar to those observed by Francis in the mid-20th century [1], [2]. These earlier studies were primarily focused on H1N1 and, to a lesser extent, H2N2 subtypes. Hence, the patterns seen here are not unique to H3N2 influenza. However, earlier authors did not have modern statistical tools and were unable to characterize the phenomena in the same detail as the present work. In addition, Francis and others were primarily focused on the primacy of the first strain to which an individual was exposed (true original antigenic sin), and did not identify the importance of age at exposure to later strains.

We propose that the age dependence observed in this study is more properly called “antigenic seniority” rather than “original antigenic sin”, as it is not only the first strain circulating in an individual's lifetime for which there is an elevated response. We find evidence that the earlier in life that someone is potentially exposed to a strain the higher their antibody titers are likely to be. In the strict interpretation of original antigenic sin, the first childhood influenza infection gains a privileged spot in the immune response, muting the immune response to later viruses and being boosted by later infections. [1] We hypothesize that antigenic seniority may work in a similar manner: viruses to which an individual is exposed early in life can be thought of as taking on senior positions in the hierarchy of immune response, each subsequent infection taking on the next most senior position. Later infections both boost the antibody response to the more senior virus and may have a lessened antibody response themselves. This hypothesis is consistent with the patterns observed in the present study and experimental evidence [3], [12], [13], though more recent work has shown that multiple immunizations can produce a broadly protective immune response [18]–[20]. Even if immune boosting and inhibition are the predominant drivers of the patterns seen, factors such as difference in influenza exposure by age likely still play a role.

## Materials and Methods

### Ethics statement

All study protocols and instruments were approved by the following institutional review boards: Johns Hopkins Bloomberg School of Public Health, University of Liverpool, University of Hong Kong, Peoples Number 12 Hospital Guangzhou, and Shantou University. Written informed consent was obtained from all participants over 12 years of age. Verbal assent was obtained for participants of 12 years of age or younger. Written permission of a legally authorized representative was obtained for all participants under the age of 18.

### Participant recruitment

Participants were enrolled from 100 randomly selected households from five study locations (20 per location) in a transect extending to the northeast from Guangzhou, China, as described in Lessler et al [21]. All household members over two years of age were eligible to participate. Household members agreeing to participate were administered informed consent and offered two levels of participation: (1) completing a questionnaire and (2) completing the questionnaire and providing a blood sample. Enrollment ran from July 8, 2009 to September 21, 2009.

### Strain selection

Nine strains of influenza A (H3N2) spanning the history of the virus from its emergence in 1968 until the present were selected for serological testing (Figure S7). We chose six vaccine strains from every second antigenic cluster, starting with a Hong Kong 1968 [10]: A/Hong Kong/1/1968 (H3N2), A/Victoria/3/1975 (H3N2), A/Bangkok/1/1979 (H3N2), A/Beijing/353/1989 (H3N2), A/Wuhan/359/1995 (H3N2) and A/Fujian/411/2002 (H3N2). In addition, three recently circulating H3N2 viruses isolated in southern China were selected for testing: A/Shantou/90/2003, A/Shantou/806/2005 and A/Shantou/904/2008. Shantou strains are genetically similar to contemporaneous vaccine strains, and may be presumed to be in same antigenic cluster as these viruses (see Figure S7).

### Serological assays

Hemagglutination inhibition (HI) and virus neutralization (NT) assays were performed for each of the nine selected strains of influenza A (H3N2) as described in Lessler et al. [21] Antibody titers were determined by testing serial two-fold dilutions from 1∶10 to 1∶1280 in duplicate (uncertain results were resolved by repeated testing in quadruplicate). Positive and negative control sera were also tested. The highest dilution resulting in complete protection of the cell monolayer in more than two of the quadruplicate wells (or both duplicate wells) was regarded as the antibody titer.

### Analysis

The effects of both participant age at time of testing and participant age in the year of strain isolation on NT titers were considered. In all cases serological results were assumed to be exact and participants with undetectable titers (<1∶10) were assumed to have a titer of 1∶5. Models capturing two hypotheses were compared: (A) the age dependency of serological response is common across strains and based only on the age at time of testing and age at time of strain isolation, and (B) the age dependency of serological response is strain specific. Generalized additive models representing each hypothesis were fit to log-neutralization data and compared using Bayesian information criteria (BIC) [22], [23].

Generalized additive models provide a flexible and integrated framework for fitting non-linear relationships between data (i.e., models with a spline term). [23] BIC heavily favors more parsimonious models, and we selected it as the primary comparison criteria to avoid over-fitting. However, we also compare models on the basis of other information based and cross-validation based criteria (e.g., cross-validated MSE and AICc). Confidence intervals in figures were created from standard errors calculated using the mgcv package in R, which are based upon the Bayesian posterior covariance matrix [23].

All analyses were repeated using HI titers yielding qualitatively identical results (Figure S8). Details of statistical models are available in supplemental Text S1. Data used in this analysis is available in Dataset S1.

All statistical analyses were performed using the R statistical package (R 2.11, www.cran.org).

## Supporting Information

Dataset S1 Individual-level data used in the analyses presented in this paper. The file contains 1,359 records for: age, age of the participant at time of sampling; is.vac, the number of years previously that the person has been vaccinated (1 for this year, greater than 1 year up to 2 years ago, 3 for 3 years ago, 4 for 4 years ago and 5 for 5 or more years ago, NA where the question was not answered); shift.age, the age of the participant in the year that the strain in question first circulated; titers, the neutralization titer against the strain in question; neut.against, the strain against which the titer is measured and for which shift.age is calculated; id, a unique study id for the analysis presented in here; and loc, a unique study id for the analysis presented here.
(CSV)

Figure S1 Box-plots of neutralization titers to strains of H3N2 from 1968 to 2008 by age at time of testing (left) and age at time of isolation of each of the viruses tested (right). Filled circles indicate medians, boxes indicate the inter-quartile range, whiskers indicate+/−1.5 inter-quartile ranges and open circles indicate outliers.
(EPS)

Figure S2 Heat map of the proportion of individuals at a given age at the time of testing having neutralization titers of the given value.
(PDF)

Figure S3 Analysis of model residuals, checking for normality and systematic patterns of bias. Red lines pass through first and third quantiles. Solid black lines indicate the mean of the residuals, dashed black lines are placed at+/−one standard deviation, and blue lines show LOESS curve fits to the residuals.
(PDF)

Figure S4 Comparison of residuals in a model with only age effects and a strain intercept (model A) versus a model with individual intercepts and strain specific age effects (model D), comparing the mean trend (blue line) with equality (black line). For most predictions model D does not systematically outperform model A, and they have similar error structures.
(PDF)

Figure S5 Model with shared age effects and strain intercepts (model A) fit to only those individuals reporting that they never have been vaccinated.
(PDF)

Figure S6 Model components with the titers to the indicated strain left out of the fitting process. Shaded areas indicate the 95% confidence interval on the spline terms from the full model. Strains have shared distributions for the effect of (A) age at time of testing (range 10 to 80, data ranges from 7 to 81) and (B) age at the time when the given strain was isolated (range −30 to 80, data range −34 to 80). Each strain has an independent intercept (C).
(PDF)

Figure S7 Timeline and genetic distance of H3N2 viruses selected for testing (in blue), and other antigenically representative H3N2 viruses (in black). Genetic distance was measured using Kimura's 2-parameters distance rescaled to a single dimension using multi-dimensional scaling. [24], [25]
(PDF)

Figure S8 Strain independent model results for HI titers. Strains have shared distributions for the effect of (A) age at time of testing (range 10 to 80, data ranges from 7 to 81) and (B) age at the time when the given strain was isolated (range −30 to 80, data range −34 to 80). Each strain has an independent intercept (C).
(PDF)

Table S1 Characteristics and performance of models of titer response, including effective degrees of freedom (DF), log-likelihood, Bayesian information criteria (BIC), corrected Akaike information criteria (AICc), mean squared error on the fit data (MSE), hold-one-out cross validated MSE, and bootstrapped average MSE (500 bootstrap iterations). Models were fit using the mgcv package in the R statistical language. In the strain independent model (A), smooth functions of age at time of testing and age at time of strain circulation were modeled as having a common effect across all strains. In the strain dependent model (B), each strain is allowed an independent relationship with a smooth function of time at testing. The random intercept model (C) extends the strain independent model, allowing each individual to have an independent intercept. The random intercept model strain dependent model (D) extends the strain dependent model in the same way. Models A–D allow strain specific intercepts. Bias was within 0.005 of 0 in all tests.
(DOCX)

Table S2 Time since last vaccination versus age. While the majority of individuals have never been vaccinated in most age groups, the relationship between time since vaccination and age is significant (simulated Chi-squared p = 0.031).
(DOCX)

Table S3 Changes in model performance after holding out each strain from the fitting process in turn. The held out strain bias and mean squared error (MSE) is calculated by predicting strain titers for the held out strain using the intercept for that strain in the full model and the “age at time of testing” and “age at time of strain isolation” spline terms from the model fit without that strain (i.e., using the shape from the reduced model shifted by the strain specific intercept). Full models were obtained by calculating the MSE using residuals only from those observations included in the comparison set. While holding out A/Hong Kong/1968 results in a model that has poor performance on that strain, its inclusion does not substantively reduce model fit to other strains.
(DOCX)

Table S4 Changes in model performance after holding out each location from the fitting process in turn.
(DOCX)

Text S1 Additional details for statistical methods.
(DOCX)
