# Supplementary figures and images for: Evidence for Antigenic Seniority in Influenza A (H3N2) Antibody Responses in Southern China
Source: PLoS Pathog. 2012 Jul 19;8(7):e1002802. doi: 10.1371/journal.ppat.1002802 (PMC3400560; doi:10.1371/journal.ppat.1002802)

titer

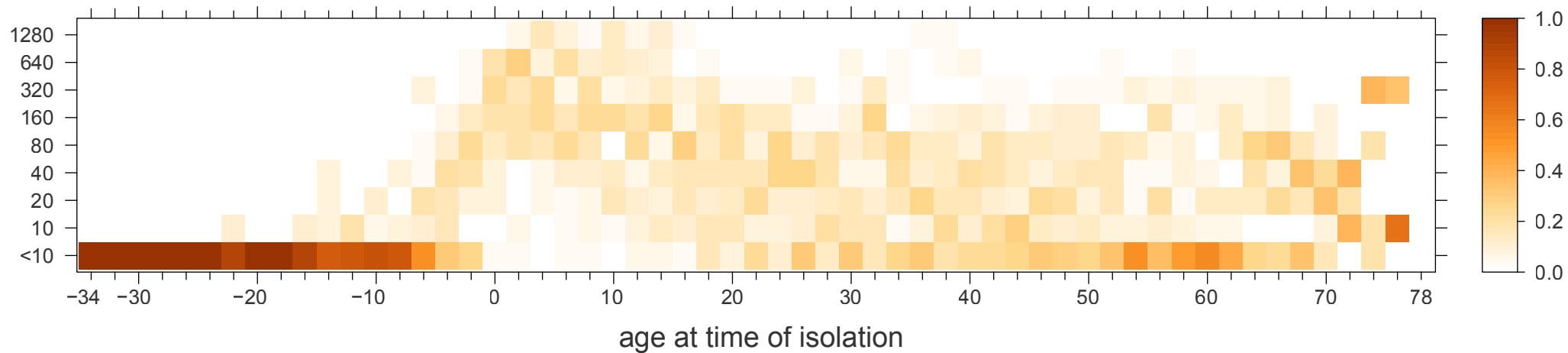

Supplement: Figure S2 — Heat map of the proportion of individuals at a given age at the time of testing having neutralization titers of the given value. (PDF) [file ppat.1002802.s003.pdf]

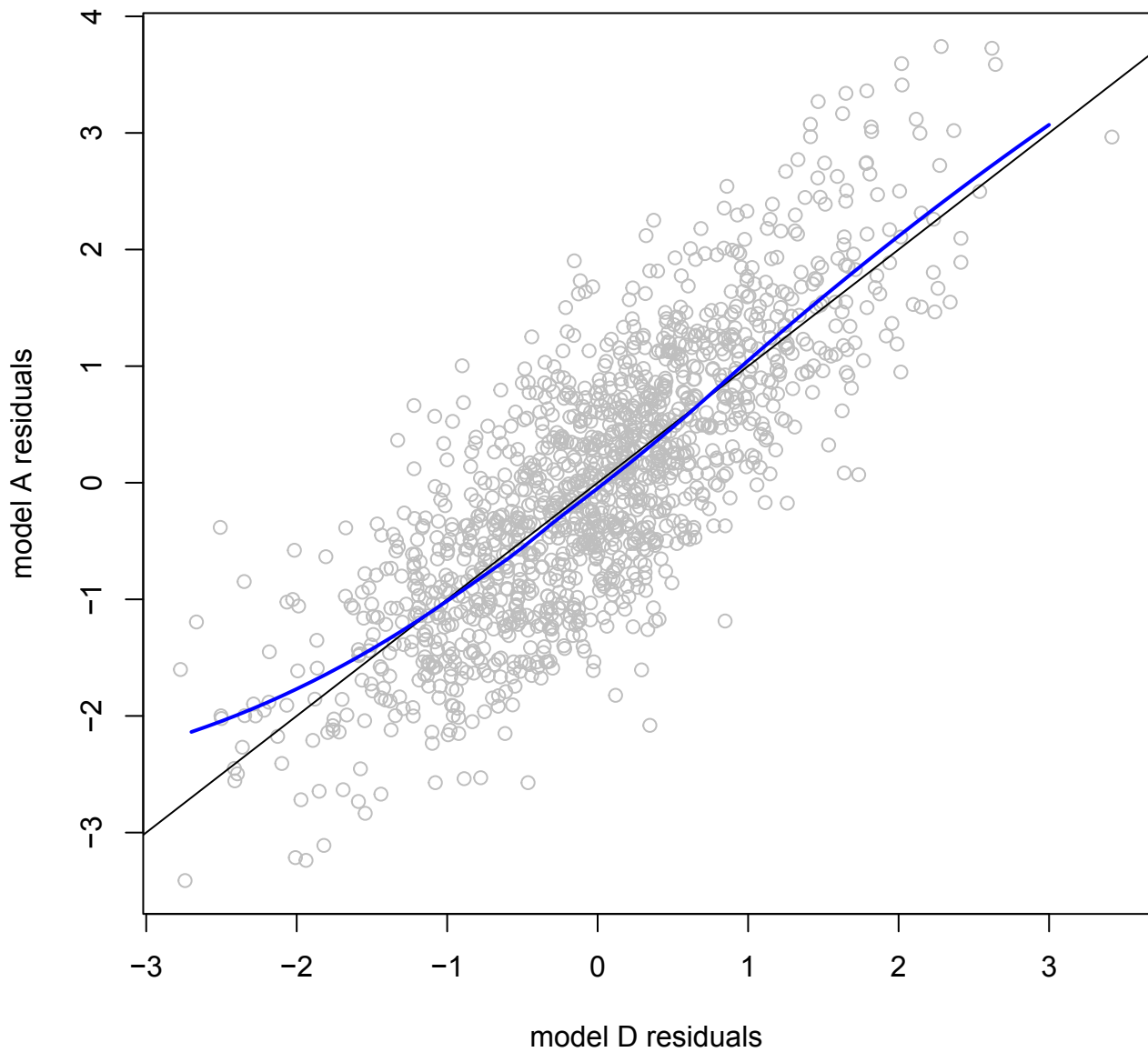

Supplement: Figure S4 — Comparison of residuals in a model with only age effects and a strain intercept (model A) versus a model with individual intercepts and strain specific age effects (model D), comparing the mean trend (blue line) with equality (black line). For most predictions model D does not systematically outperform model A, and they have similar error structures. (PDF) [file ppat.1002802.s005.pdf]

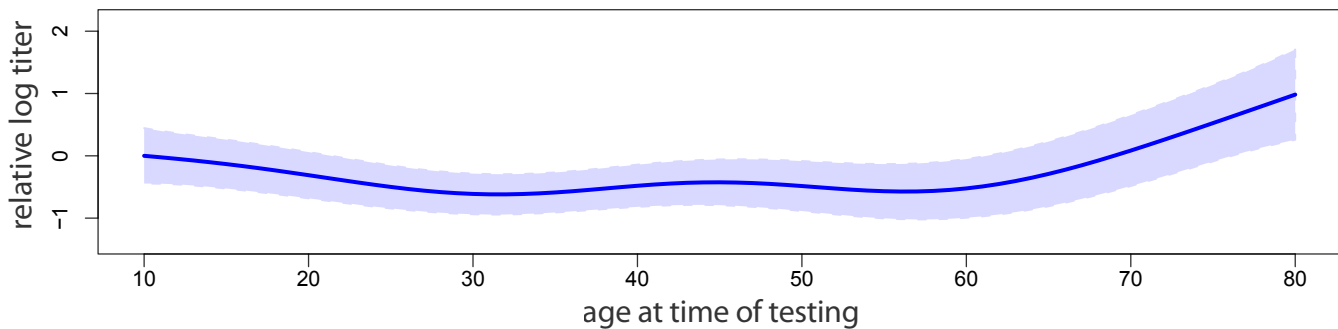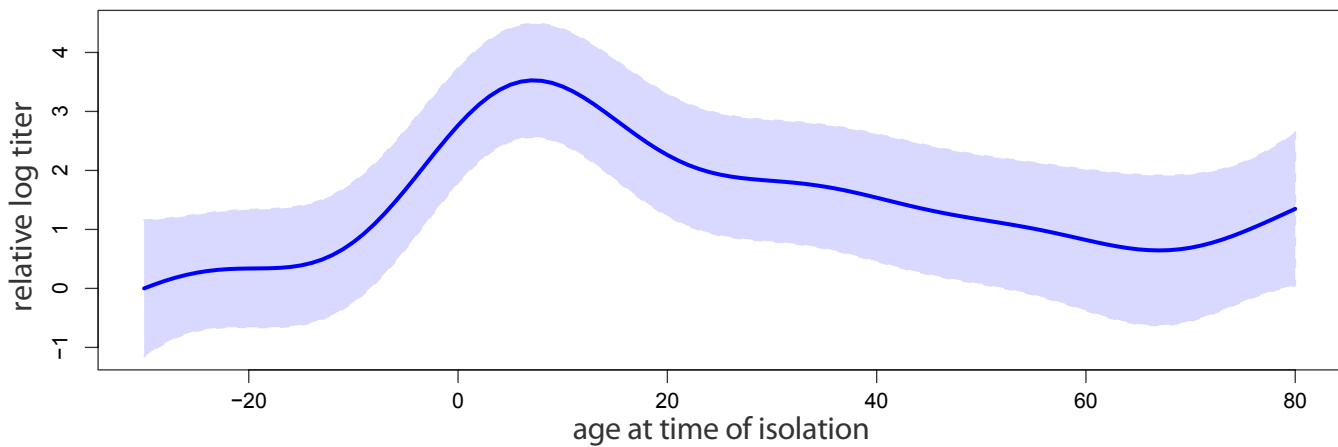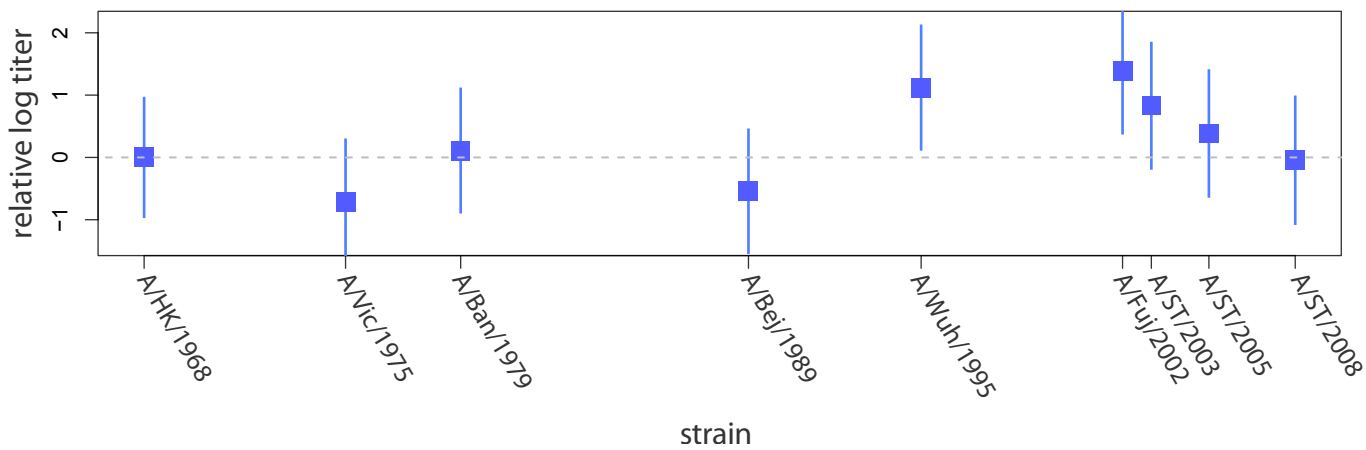

Supplement: Figure S5 — Model with shared age effects and strain intercepts (model A) fit to only those individuals reporting that they never have been vaccinated. (PDF) [file ppat.1002802.s006.pdf]

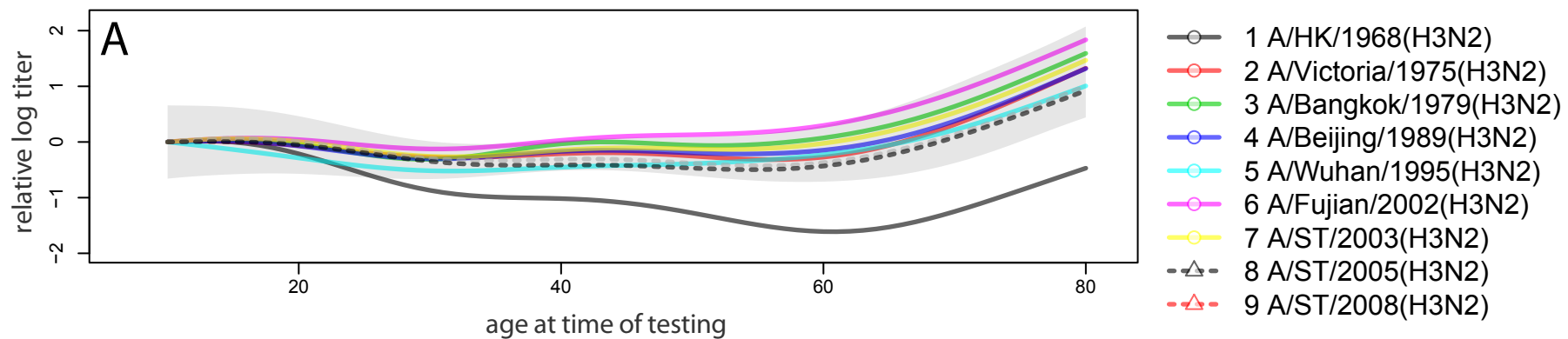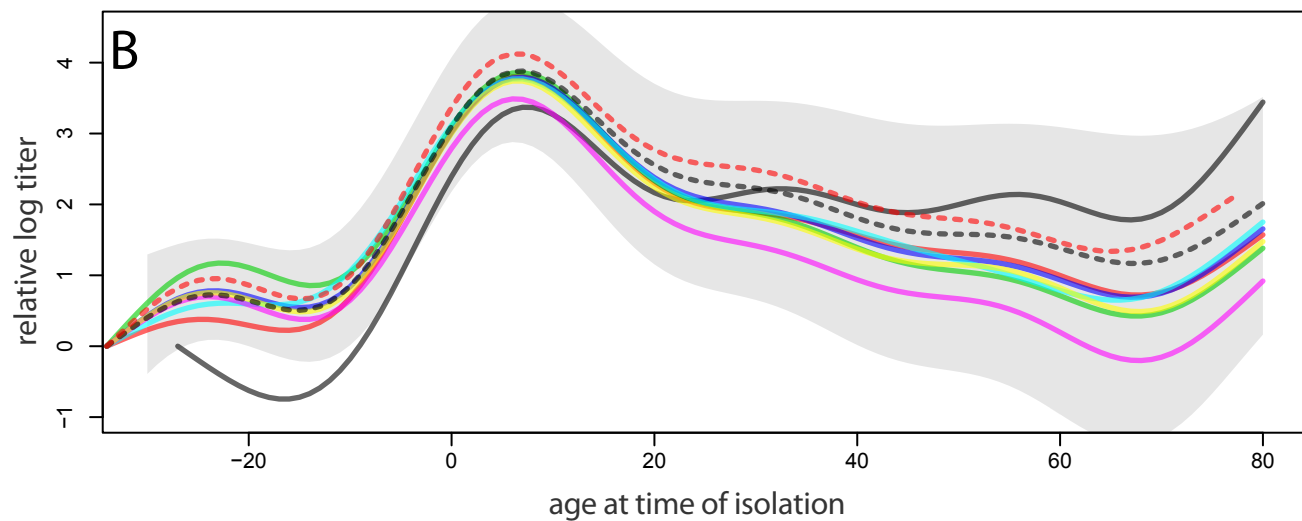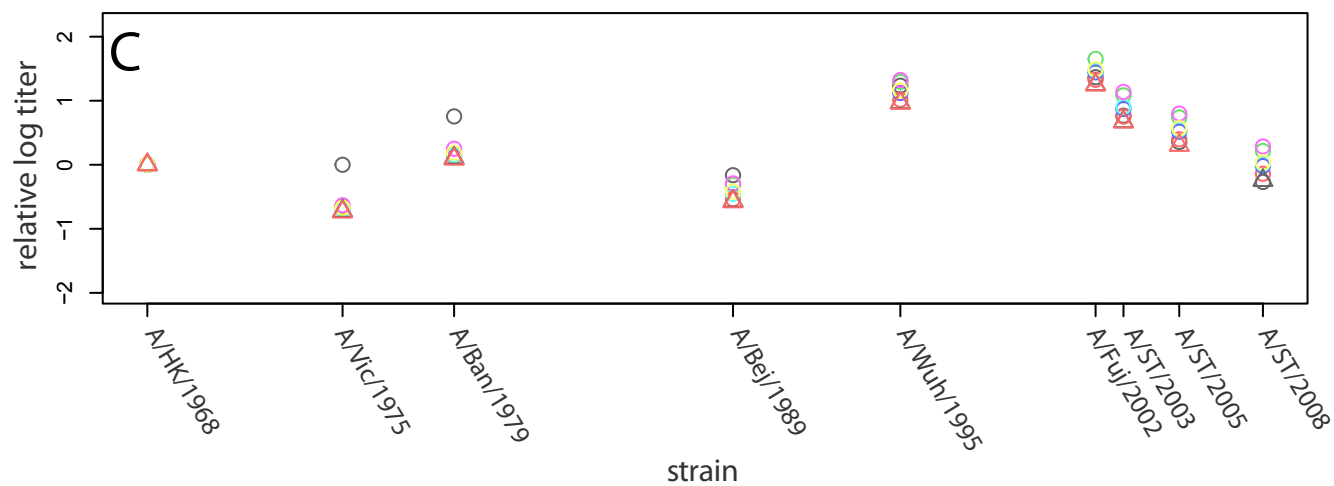

Supplement: Figure S6 — Model components with the titers to the indicated strain left out of the fitting process. Shaded areas indicate the 95% confidence interval on the spline terms from the full model. Strains have shared distributions for the effect of (A) age at time of testing (range 10 to 80, data ranges from 7 to 81) and (B) age at the time when the given strain was isolated (range −30 to 80, data range −34 to 80). Each strain has an independent intercept (C). (PDF) [file ppat.1002802.s007.pdf]

genetic distance

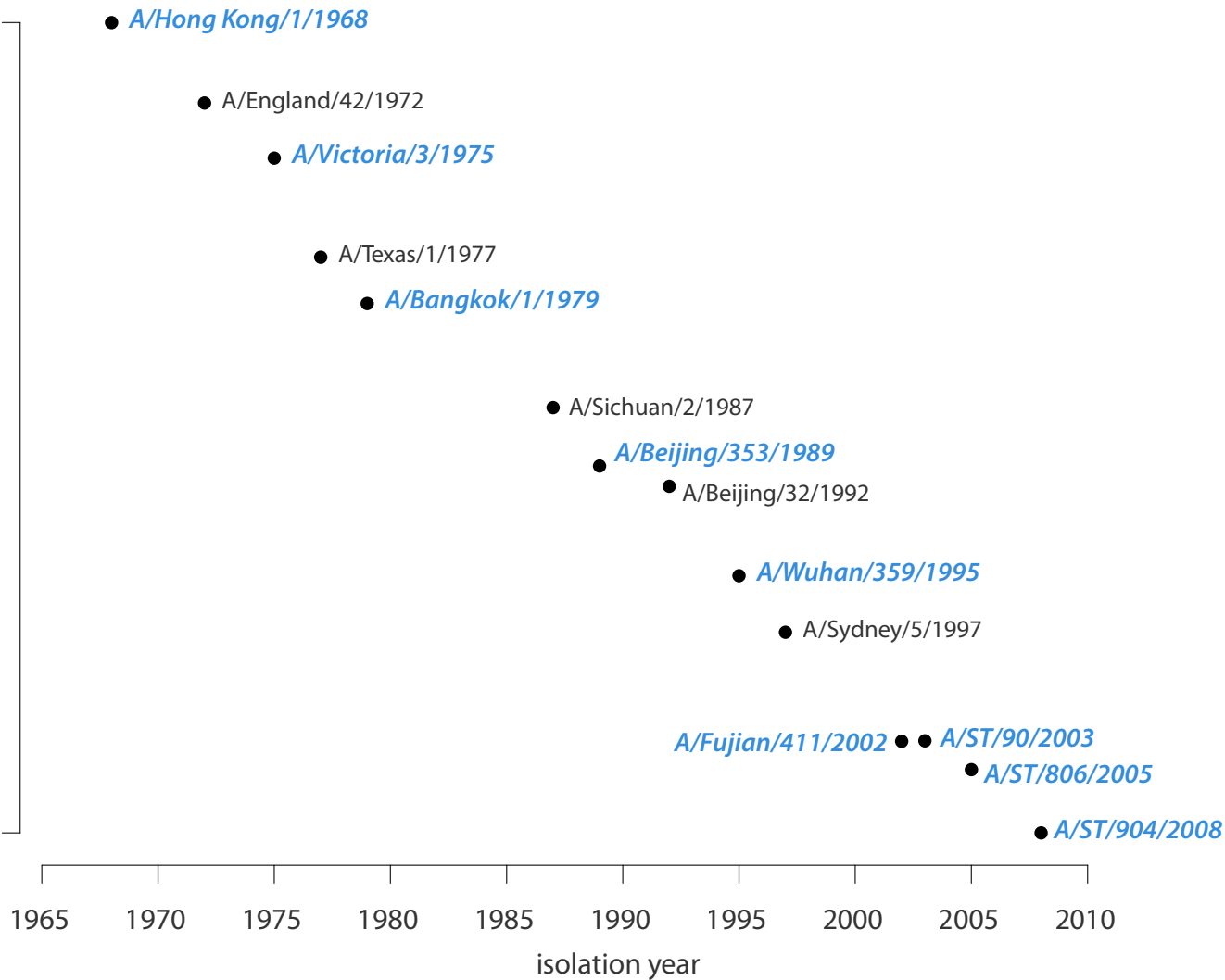

Supplement: Figure S7 — Timeline and genetic distance of H3N2 viruses selected for testing (in blue), and other antigenically representative H3N2 viruses (in black). Genetic distance was measured using Kimura's 2-parameters distance rescaled to a single dimension using multi-dimensional scaling. [24], [25] (PDF) [file ppat.1002802.s008.pdf]

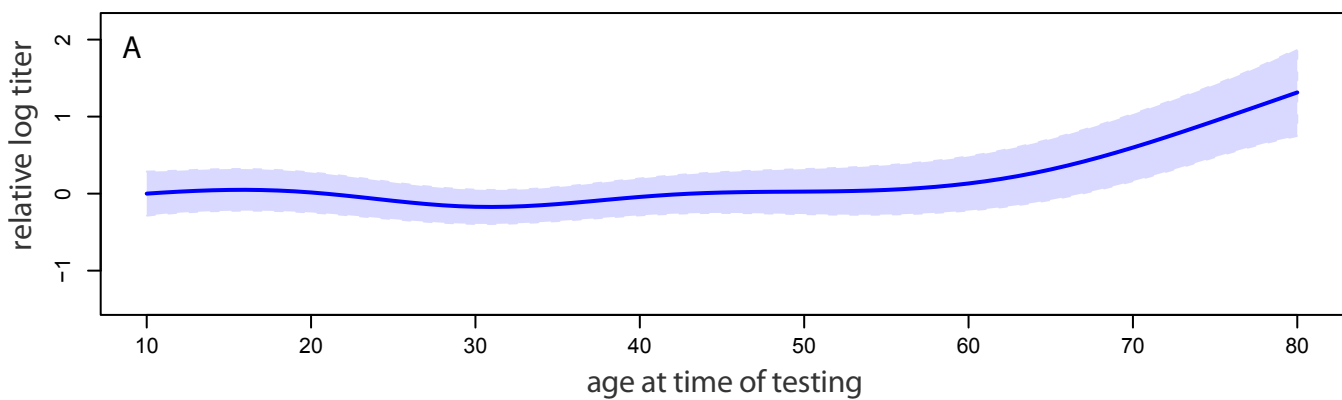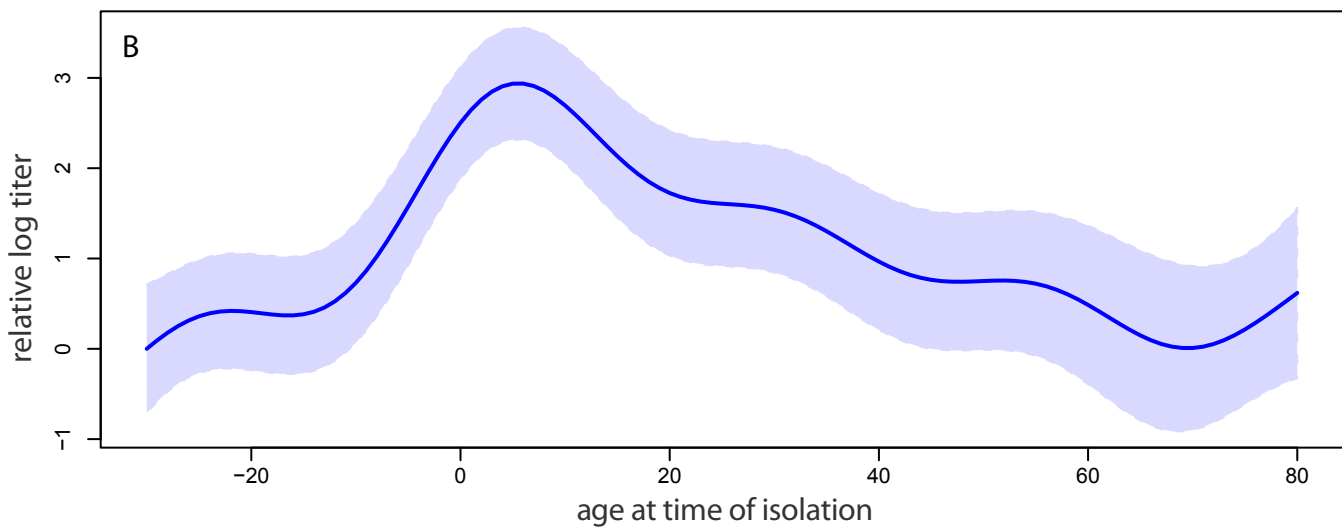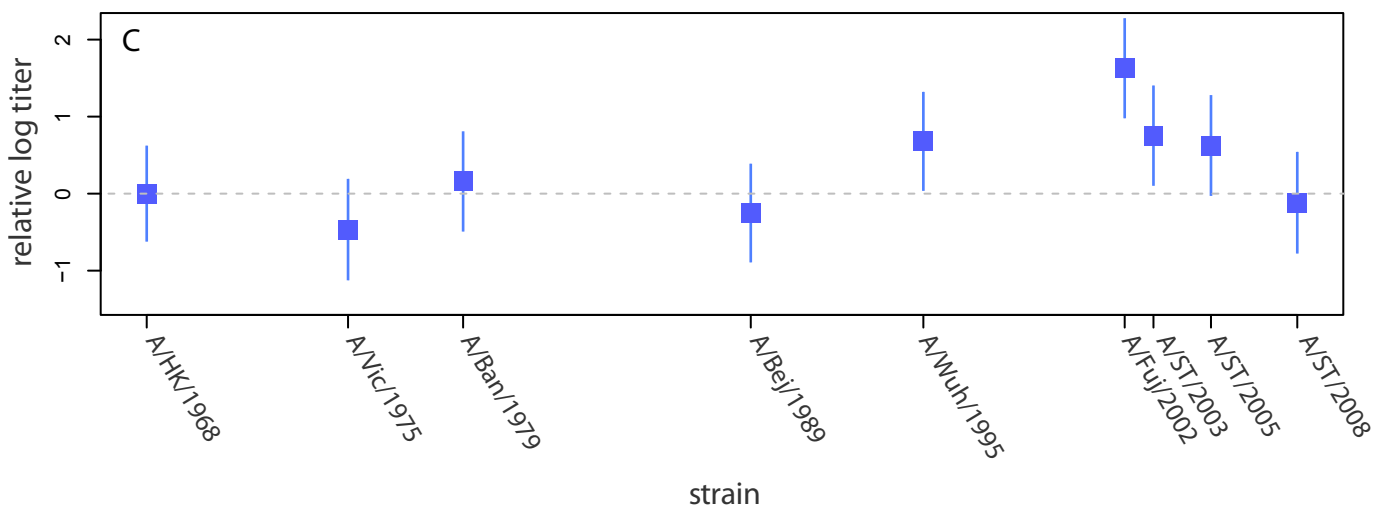

Supplement: Figure S8 — Strain independent model results for HI titers. Strains have shared distributions for the effect of (A) age at time of testing (range 10 to 80, data ranges from 7 to 81) and (B) age at the time when the given strain was isolated (range −30 to 80, data range −34 to 80). Each strain has an independent intercept (C). (PDF) [file ppat.1002802.s009.pdf]
