# Supplementary material for: Evidence for Antigenic Seniority in Influenza A (H3N2) Antibody Responses in Southern China
Source: PLoS Pathog. 2012 Jul 19;8(7):e1002802. doi: 10.1371/journal.ppat.1002802 (PMC3400560; doi:10.1371/journal.ppat.1002802)
Supplement: Figure S3 — Analysis of model residuals, checking for normality and systematic patterns of bias. Red lines pass through first and third quantiles. Solid black lines indicate the mean of the residuals, dashed black lines are placed at+/−one standard deviation, and blue lines show LOESS curve fits to the residuals. (PDF) [file ppat.1002802.s004.pdf]

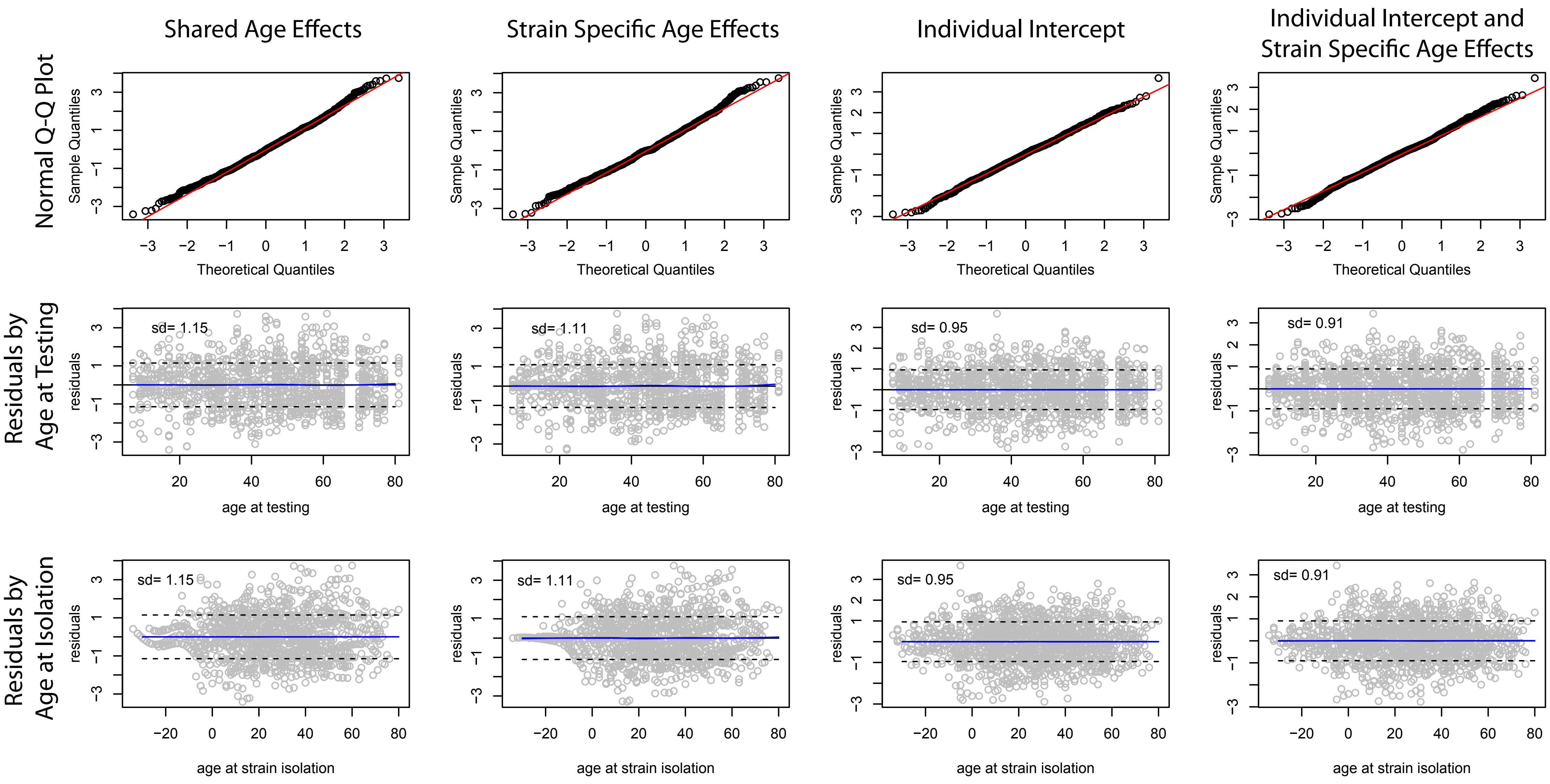

Shared Age Effects
Strain Specific Age Effects
Individual Intercept
Individual Intercept and Strain Specific Age Effects
Normal Q-Q Plot
Sample Quantiles
Theoretical Quantiles
Residuals by Age at Testing
residuals
age at testing
sd= 1.15
sd= 1.11
sd= 0.95
sd= 0.91
Residuals by Age at Isolation
age at strain isolation
