# Supplementary material for: Evidence for Antigenic Seniority in Influenza A (H3N2) Antibody Responses in Southern China
Source: PLoS Pathog. 2012 Jul 19;8(7):e1002802. doi: 10.1371/journal.ppat.1002802 (PMC3400560; doi:10.1371/journal.ppat.1002802)
Supplement: Table S1 — Characteristics and performance of models of titer response, including effective degrees of freedom (DF), log-likelihood, Bayesian information criteria (BIC), corrected Akaike information criteria (AICc), mean squared error on the fit data (MSE), hold-one-out cross validated MSE, and bootstrapped average MSE (500 bootstrap iterations). Models were fit using the mgcv package in the R statistical language. In the strain independent model (A), smooth functions of age at time of testing and age at time of strain circulation were modeled as having a common effect across all strains. In the strain dependent model (B), each strain is allowed an independent relationship with a smooth function of time at testing. The random intercept model (C) extends the strain independent model, allowing each individual to have an independent intercept. The random intercept model strain dependent model (D) extends the strain dependent model in the same way. Models A–D allow strain specific intercepts. Bias was within 0.005 of 0 in all tests. (DOCX) [file ppat.1002802.s010.docx]

| ***Model*** | ***Effective DF*** | ***Log Likelihood*** | ***BIC*** | ***AICc*** | ***Fit Data***  ***MSE*** | ***Cross Val.***  ***MSE*** | ***Bootstrap***  ***Avg. MSE*** |
| --- | --- | --- | --- | --- | --- | --- | --- |
| (A) Strain Independent | 23.4 | -2114.4 | **4397.3** | 4276.3 | 1.32 | 1.36 | 1.33 |
| (B) Strain Dependent | 65.0 | -2067.3 | 4603.7 | 4271.3 | 1.23 | 1.36 | 1.28 |
| (C) Random Intercept | 167.8 | -1864.7 | 4940.2 | 4112.3 | 0.91 | 1.18 | --- |
| (D) Strain Dependent +  Random Intercept | 203.0 | -1793.6 | 5051.6 | **4064.9** | 0.82 | 1.16 | **---** |

**Table S1:** Characteristics and performance of models of titer response, including effective degrees of freedom (DF), log-likelihood, Bayesian information criteria (BIC), corrected Akaike information criteria (AICc), mean squared error on the fit data (MSE), hold-one-out cross validated MSE, and bootstrapped average MSE (500 bootstrap iterations). Models were fit using the mgcv package in the R statistical language. In the strain independent model (A), smooth functions of age at time of testing and age at time of strain circulation were modeled as having a common effect across all strains. In the strain dependent model (B), each strain is allowed an independent relationship with a smooth function of time at testing. The random intercept model (C) extends the strain independent model, allowing each individual to have an independent intercept. The random intercept model strain dependent model (D) extends the strain dependent model in the same way. Models A-D allow strain specific intercepts. Bias was within 0.005 of 0 in all tests.
