# Supplementary material for: Evidence for Antigenic Seniority in Influenza A (H3N2) Antibody Responses in Southern China
Source: PLoS Pathog. 2012 Jul 19;8(7):e1002802. doi: 10.1371/journal.ppat.1002802 (PMC3400560; doi:10.1371/journal.ppat.1002802)
Supplement: Table S2 — Time since last vaccination versus age. While the majority of individuals have never been vaccinated in most age groups, the relationship between time since vaccination and age is significant (simulated Chi-squared p = 0.031). (DOCX) [file ppat.1002802.s011.docx]

|  | **Time Since Last Vaccination** | | | | | |
| --- | --- | --- | --- | --- | --- | --- |
| **Age** | *<1 year* | *1 year* | *2-5 years* | *>5 years* | *Never* | *Unsure/*  *Unknown* |
| *<10* | 1 | 0 | 1 | 0 | 1 | 2 |
| *10-19* | 2 | 1 | 2 | 1 | 8 | 2 |
| *20-39* | 1 | 0 | 1 | 11 | 25 | 6 |
| *40-59* | 1 | 0 | 2 | 6 | 38 | 6 |
| *60+* | 1 | 0 | 0 | 1 | 28 | 3 |
| ***Total*** | 6 | 1 | 6 | 19 | 100 | 19 |
