# Supplementary material for: Evidence for Antigenic Seniority in Influenza A (H3N2) Antibody Responses in Southern China
Source: PLoS Pathog. 2012 Jul 19;8(7):e1002802. doi: 10.1371/journal.ppat.1002802 (PMC3400560; doi:10.1371/journal.ppat.1002802)
Supplement: Table S3 — Changes in model performance after holding out each strain from the fitting process in turn. The held out strain bias and mean squared error (MSE) is calculated by predicting strain titers for the held out strain using the intercept for that strain in the full model and the “age at time of testing” and “age at time of strain isolation” spline terms from the model fit without that strain (i.e., using the shape from the reduced model shifted by the strain specific intercept). Full models were obtained by calculating the MSE using residuals only from those observations included in the comparison set. While holding out A/Hong Kong/1968 results in a model that has poor performance on that strain, its inclusion does not substantively reduce model fit to other strains. (DOCX) [file ppat.1002802.s012.docx]

**Table S3:**  Changes in model performance after holding out each strain from the fitting process in turn. The held out strain bias and mean squared error (MSE) is calculated by predicting strain titers for the held out strain using the intercept for that strain in the full model and the “age at time of testing” and “age at time of strain isolation” spline terms from the model fit without that strain (i.e., using the shape from the reduced model shifted by the strain specific intercept). Full models were obtained by calculating the MSE using residuals only from those observations included in the comparison set. While holding out A/Hong Kong/1968 results in a model that has poor performance on that strain, its inclusion does not substantively reduce model fit to other strains.

|  | ***Held out Strain*** | | | | ***Remaining Strains*** | | |
| --- | --- | --- | --- | --- | --- | --- | --- |
| *Held out Strain* | *Bias* | *MSE* | *Full Model MSE* | *Relative MSE* | *MSE* | *Full Model MSE* | *Relative MSE* |
| **A/HK/1968** | **-0.60** | **1.43** | **0.84** | **1.70** | **1.37** | **1.37** | **0.996** |
| A/Victoria/1975 | -0.03 | 1.18 | 1.09 | 1.08 | 1.34 | 1.34 | 0.997 |
| A/Bangkok/1979 | 0.13 | 1.19 | 1.11 | 1.07 | 1.34 | 1.34 | 0.996 |
| A/Beijing/1989 | 0.02 | 1.09 | 1.07 | 1.01 | 1.34 | 1.35 | >0.999 |
| A/Wuhan/1995 | 0.01 | 1.66 | 1.50 | 1.10 | 1.29 | 1.29 | 0.997 |
| A/Fujian/2002 | -0.11 | 1.64 | 1.60 | 1.03 | 1.28 | 1.28 | >0.999 |
| A/ST/2003 | -0.05 | 1.50 | 1.49 | 1.01 | 1.29 | 1.29 | >0.999 |
| A/ST/2005 | 0.05 | 1.88 | 1.84 | 1.02 | 1.25 | 1.25 | 0.999 |
| A/ST/2008 | 0.05 | 1.36 | 1.29 | 1.05 | 1.32 | 1.32 | 0.998 |
