# Supplementary material for: Evidence for Antigenic Seniority in Influenza A (H3N2) Antibody Responses in Southern China
Source: PLoS Pathog. 2012 Jul 19;8(7):e1002802. doi: 10.1371/journal.ppat.1002802 (PMC3400560; doi:10.1371/journal.ppat.1002802)
Supplement: Table S4 — Changes in model performance after holding out each location from the fitting process in turn. (DOCX) [file ppat.1002802.s013.docx]

**Table S4:**  Changes in model performance after holding out each location from the fitting process in turn.

|  | ***Held out Location*** | | | | ***Remaining Locations*** | | |
| --- | --- | --- | --- | --- | --- | --- | --- |
| *Held out Location* | *Bias* | *MSE* | *Full Model MSE* | *Relative MSE* | *MSE* | *Full Model MSE* | *Relative MSE* |
| Location 1 | 0.15 | 1.35 | 1.26 | 1.07 | 1.31 | 1.33 | 0.99 |
| Location 2 | 0.08 | 1.49 | 1.46 | 1.02 | 1.28 | 1.29 | 0.997 |
| Location 3 | -0.15 | 1.68 | 1.55 | 1.09 | 1.25 | 1.27 | 0.99 |
| Location 4 | 0.04 | 1.25 | 1.18 | 1.05 | 1.37 | 1.37 | 0.996 |
| Location 5 | -0.17 | 1.29 | 1.22 | 1.05 | 1.33 | 1.33 | 0.998 |
