# Supplementary material for: Evidence for Antigenic Seniority in Influenza A (H3N2) Antibody Responses in Southern China
Source: PLoS Pathog. 2012 Jul 19;8(7):e1002802. doi: 10.1371/journal.ppat.1002802 (PMC3400560; doi:10.1371/journal.ppat.1002802)
Supplement: Text S1 — Additional details for statistical methods. (DOCX) [file ppat.1002802.s014.docx]

# Text S1: Detailed Statistical Methods

## Models of Age and Titer

The serological response was modeled as a function of age and strain using three models detailed below.

*Model A: Strain Independent*

This model captures the hypothesis that the relationship between age and measured NT titer is independent of strain, and a function only of the age at time of testing and age when the strain was isolated. Strains are allowed to have independent intercepts.

$$\log y_{ij}=\alpha_{j}+\beta S\left( x_{i} \right)+\gamma S^{'}(x_{i}-q_{j})$$

where $y_{ij}$ is the neutralization titer of person $i$ to strain $j$, $\alpha_{j}$ is a strain specific intercept, $\beta$ and $\gamma$ are vectors of spline coefficients,  is the age of the participant at the time of testing, $q_{j}$ is the years since strain $j$ was isolated, and $S\left( a \right)$ is the value at age $a$ of a smoothing spline basis.

*Model B: Strain Dependent*

This model captures the hypothesis that the way in which age is associated with measured NT titers is strain specific. That is the relationship will follow different functional forms for different strains.

$$\log y_{ij}=\alpha_{j}+\beta_{j}S\left( x_{i} \right)$$

where $y_{ij}$is the neutralization titer of person $i$ to strain$j$, $\alpha_{j}$ is a strain specific intercept, $\beta_{j}$ is the vector of strain specific spline coefficients, and $S\left( x_{i} \right)$ is the value at age $x_{i}$ of the basis of a smoothing spline on age.

*Model C: Strain Independent with Individual Random Intercept*

This model captures the same hypothesis as model A, but attempts to control for any person to person differences in neutralization response not accounted for by age.

$$\log y_{ij}=\alpha_{j}+\delta_{i}+\gamma S(x_{i}-q_{j})$$

where $y_{ij}$ is the neutralization titer of person $i$ to strain $j$, $\alpha_{j}$ is a strain specific intercept, $\delta_{i}$ is an individual specific intercept, $\gamma$ is a vector of spline coefficients, $x_{i}$ is the age of participant $i$ at the time of testing, $q_{j}$ is the time since strain $j$ was isolated, and $S\left( a \right)$ is the value at age $a$ of a smoothing spline basis.

*Model D: Strain Dependent with Individual Random Intercept*

This model captures the same hypothesis as model B, but attempts to control for any person to person differences in neutralization response not accounted for by age.

$$\log y_{ij}=\alpha_{j}+\delta_{i}+\beta_{j}S(x_{i})$$

where $y_{ij}$ is the neutralization titer of person $i$ to strain $j$, $\alpha_{j}$ is a strain specific intercept, $\delta_{i}$ is an individual specific intercept, $\beta_{j}$ is the vector of strain specific spline coefficients, and $S\left( x_{i} \right)$ is the value at age $x_{i}$ of the basis of a smoothing spline on age.

*Model Fitting and Evaluation:*

Within each model type, models were fit using the gam function in the mgcv package for R (cran.r-project.org/web/packages/mgcv/). Comparison between models were made based on Bayesian information criteria (BIC), corrected Akaike information criteria (AICc) and cross validation tests.

## Models of Original Antigenic Sin

We fit four models capturing different forms of the hypothesis that the observed patterns emerged from cross reactivity and classical original antigenic sin (OAS). These models were fit to log neutralization titers to A/Hong Kong/1968, A/Victoria/1975, A/Bangkok/1979, A/Beijing/1989, A/Wuhan/1995 and A/Fujian/2002. Models were compared to Model A from the main analysis fit to the same subset of the data.

*OAS Model 1 (relative BIC 80.6)*

Captures the hypothesis that observed titers are based solely on cross reactivity with the first possibly infecting strain:

$$\log y_{ij}=\alpha_{j}+\beta d_{ij}$$

where $y_{ij}$ is the neutralization titer of person $i$ to strain $j$, $\alpha_{j}$ is a strain specific intercept, $d_{ij}$ is the antigenic distance between strain $j$ and the first possible infecting strain for person $i$, and $\beta$ is a model coefficient.

*OAS Model 2 (relative BIC 34.3)*

Captures the hypothesis that observed titers are based on cross reactivity with the first possibly infecting strain and whether someone was alive at the time of circulation:

$$\log y_{ij}=\alpha_{j}+\beta d_{ij}+\gamma l_{ij}$$

where $y_{ij}$ is the neutralization titer of person $i$ to strain $j$, $\alpha_{j}$ is a strain specific intercept, $d_{ij}$ is the antigenic distance between strain $j$ and the first possible infecting strain for person $i$, $l_{ij}$ is an indicator of whether participant $i$ when strain $j$ circulated, and $\beta$ and $\gamma$ are model coefficients.

*OAS Model 3 (relative BIC 82.8)*

Captures the hypothesis that observed titers are based solely on cross reactivity with the first possibly infecting strain, allowing more flexibility in cross reactivity:

$$\log y_{ij}=\alpha_{j}+\boldsymbol{\beta}S(d_{ij})$$

where $y_{ij}$ is the neutralization titer of person $i$ to strain $j$, $\alpha_{j}$ is a strain specific intercept, $S(d_{ij})$ is a spline of the antigenic distance between strain $j$ and the first possible infecting strain for person $i$, and $\boldsymbol{\beta}$ is the vector of spline coefficients.

*OAS Model 4 (relative BIC 34.3)*

Captures the hypothesis that observed titers are based on cross reactivity with the first possibly infecting strain and whether someone was alive at the time of circulation, allowing more flexibility in cross reactivity:

$$\log y_{ij}=\alpha_{j}+\boldsymbol{\beta} S(d_{ij})+\gamma l_{ij}$$

where $y_{ij}$ is the neutralization titer of person $i$ to strain $j$, $\alpha_{j}$ is a strain specific intercept, $S(d_{ij})$ is a spline of the antigenic distance between strain $j$ and the first possible infecting strain for person $i$, $\boldsymbol{\beta}$ is the vector of spline coefficients, $l_{ij}$ is an indicator of whether participant $i$ when strain $j$ circulated, and $\gamma$

is a model coefficient. Note that the best fit version of this model is identical to OAS model 2 (i.e., the spline is linear).
